# Exploring the determinants of health and wellbeing in communities living in proximity to coal seam gas developments in regional Queensland

**DOI:** 10.1186/s12889-017-4568-1

**Published:** 2017-08-03

**Authors:** Fiona Mactaggart, Liane McDermott, Anna Tynan, Christian A. Gericke

**Affiliations:** 1 Department of Health Care Management, Berlin University of Technology, Berlin, Germany; 2 Queensland Centre for Domestic and Family Violence Research, School of Nursing and Midwifery, CQ University Australia, Brisbane, Australia; 3 University of Queensland School of Public Health, Brisbane, Australia; 4 Anton Breinl Centre for Health Systems Strengthening, Colleges of Medicine and Public Health, James Cook University, Cairns, Australia

**Keywords:** Health needs assessment, Wellbeing, Rural health, Social determinants of health

## Abstract

**Background:**

There is some concern that coal seam gas mining may affect health and wellbeing through changes in social determinants such as living and working conditions, local economy and the environment. The onward impact of these conditions on health and wellbeing is often not monitored to the same degree as direct environmental health impacts in the mining context, but merits attention. This study reports on the findings from a recurrent theme that emerged from analysis of the qualitative component of a comprehensive Health Needs Assessment (HNA) conducted in regional Queensland: that health and wellbeing of communities was reportedly affected by nearby coal seam gas (CSG) development beyond direct environmental impacts.

**Methods:**

Qualitative analysis was initially completed using the Framework Method to explore key themes from 11 focus group discussions, 19 in-depth interviews, and 45 key informant interviews with health and wellbeing service providers and community members. A key theme emerged from the analysis that forms the basis of this paper. This study is part of a larger comprehensive HNA involving qualitative and quantitative data collection to explore the health and wellbeing needs of three communities living in proximity to CSG development in regional Queensland, Australia.

**Results:**

Communities faced social, economic and environmental impacts from the rapid growth of CSG development, which were perceived to have direct and indirect effects on individual lifestyle factors such as alcohol and drug abuse, family relationships, social capital and mental health; and community-level factors including social connectedness, civic engagement and trust.

**Conclusions:**

Outer regional communities discussed the effects of mining activity on the fabric of their town and community, whereas the inner regional community that had a longer history of industrial activity discussed the impacts on families and individual health and wellbeing. The findings from this study may inform future health service planning in regions affected by CSG in the development /construction phase and provide the mining sector in regional areas with evidence from which to develop social responsibility programs that encompass health, social, economic and environmental assessments that more accurately reflect the needs of the affected communities.

## Background

Regional Queensland has been a focus of Australia’s coal seam gas (CSG) development over the past decade. CSG is a natural gas that is extracted via wells drilled in to coal seams, and involves exploration of land for CSG deposits, production, transportation and distribution. Significant CSG deposits are found in Canada, China, USA, and Australia, and were first explored in regional Queensland in the late 1970’s, which led to commercial production from 2006. CSG is utilised domestically, but a proportion is converted in to liquefied natural gas (LNG) and exported internationally off the Queensland coast [1]. Growth of the CSG industry and the relatively large geographic span of exploration and extraction means that ‘mining activity’ often co-exists with primary production of some of Queensland’s most diverse agricultural land, with positive and negative implications [1]. There is anecdotal concern that the environmental, economical and social change in the community brought about by the labour intensive development stage of CSG mining can have implications for health and wellbeing [2].

### CSG development and public health

There is rich evidence of potential public health implications of extracting conventional resources like coal, diamond and oil internationally [3–5]. However, with the recent emergence of CSG development, there is less known of the potential health impacts in communities as they undergo changes in their environment [6–9]. Broader social determinants of health, like changes in working conditions, community networks or access to services could have serious implications for health and wellbeing in mining or resource settings, and are less understood [2, 10]. There is anecdotal concern that CSG development may have indirect and long-term impacts on the health of communities in which they operate but the scientific evidence is lacking [10].

Growth of CSG development has been rapid, in that approximately 1634 wells have been drilled between 2013 and 2014 alone, and reserves were being discovered at an unprecedented rate. Regional Queensland represents more than 90% of the total gas produced in the state [11]. CSG extraction often occurs on active farms and grazing properties, involving direct interaction with farmers and local community members, and there is some evidence that CSG development can bring about stress and anxiety [1]. There is also a huge demand for labour in the early stages of CSG development; these roles cannot be completely filled locally and thus large workforces often temporarily reside in ‘host communities’. Population influx and influence on community structure can impact social capital through reduced social bonds and networks and there is concern for increased risky lifestyle behaviours like drug use and alcoholism that spill over to the communities from the mine workforce [12, 13].

The following paper forms part of a larger Health Needs Assessment (HNA) research project conducted in regions were CSG development was occurring. The purpose of the larger project was not to specifically identify the direct impacts of mining activity, but rather to assess broader population-level health and wellbeing issues in the communities and explore trends and possible determinants. Health is defined as ‘a state of complete physical, mental and social wellbeing and not merely the absence of disease or infirmity’ [14]. In conjunction, wellbeing is used to describe elements of life that impact on its quality, determining an individual’s level of personal satisfaction, happiness and psychological health. Wellbeing may also include community-level factors, such as satisfaction with one’s environment, and the level of social connectedness and belonging. This study reports on the findings from a recurring theme that emerged from the qualitative component of the analysis: that health and wellbeing needs were associated with the development stage of nearby CSG mining.

The cyclical nature of mining and the unpredictability of its activity lifespan can have serious implications for surrounding communities and presents governments with the challenge of responding efficiently and effectively to evolving needs. A deeper understanding of the health and wellbeing context in mining communities is pertinent to enable community and health services to prepare for the impacts of social, environmental and economic fluctuations that might come with a mining boom or bust.

## Methods

### Theoretical framework: Health needs assessment

This study utilised an HNA model to investigate the communities of interest. HNAs are a systematic tool to explore and identify inequalities and health priorities and are useful in identifying health gaps and trends [15]. An HNA starts with a *population* rather than a project and underpinning the HNA approach is the social determinants of health framework, which describes the complex, multi-layered influencing factors, which can impact the health of an individual [15]. These factors include individual lifestyle factors, social and community networks, and the broader socio-economic, cultural and environmental conditions within which one lives. Inclusion of wellbeing indicators at an individual and community level give an indication of quality of life and satisfaction with one’s living environment [16].

### Study setting

The study was conducted in three local government areas (LGAs): A, B and C in regional Queensland, Australia during June and December 2014. The major township of LGA A, which is furthest from Queensland’s capital city of Brisbane, de-identified as Region 1, is classified as outer regional. LGA B’s two major townships, Region 2 and 3 are defined as inner and outer; and LGA C’s major township, Region 4, is defined as inner regional, according to the Accessibility/Remoteness Index of Australia (ARIA) (Table 1) [17]. ARIA criteria determines remoteness by measuring road distance to service centres, and is compared to ‘unrestricted’ accessibility in major cities. Geographical areas are categorised as major cities, inner regional, outer regional, remote or very remote. [18] ARIA is deemed an appropriate index for this research given it explores implications of the rural context and social determinants of health.

**Table 1:** Demographic and economic summaries of four study sites in regional Queensland, 2014

	Region 1	Region 2 & 3	Region 4
Demographics			
ARIA classification	Outer regional^a^	Inner and outer regional	Inner regional^b^
LGA Land Area	58,800 km^2^	38,000 km^2^	10,500 km^2^
Population	14,000	34,000	66,000
% aged <55 years	76%	74.5%^c^	80%
Economic environment			
Main industries	Mining and agriculture	Agriculture, mining and manufacturing	Mining and manufacturing
Median family income	$1444/week	$1294/week	$1941/week

^a^Significantly restricted accessibility to goods, services and opportunities for social interaction

^b^Some restricted accessibility to goods, services and opportunities for social interaction

^c^On average, population slightly older than the total Queensland population [37]

Mining activity in all of the LGAs was in the development phase during data collection in 2014, which brought a high demand for labour, mostly in the form of non-resident workers, or fly-in-fly-out (FIFO) and drive-in-drive-out (DIDO) employees who resided in the communities whilst on shift. LGA B experienced an increase in non-resident population by 9100 during 2015, compared with 5120 in LGA A. The number of businesses increased from 495 in Region 2 in 2012 to 1255 in 2013, and to 1166 in 2014. In Region 1 a similar pattern was seen but on a smaller scale (435, 790 and 755). At the time of publication, however, mining construction has drawn to a close, leading to an operational phase and a marked decrease in housing and rental prices following the out flux of FIFO and DIDO workforce.

### Study design

**Fig. 1:**
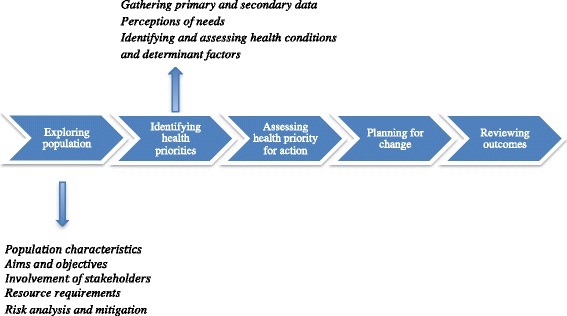
A simplified HNA process describing the first two stages used in this study. *Adapted from Cavanagh and Chadwick* [14]

Qualitative research methodology was utilised to explore the health and wellbeing needs of the communities of interest. This method was part of a larger mixed-method cross-sectional study based on the five principles of HNAs as defined by the National Institute for Health and Care Excellence (Fig. 1) [15]. The research team is preparing a further manuscript that presents quantitative results from the HNA and aims to compare and contrast with qualitative findings.

### Data collection tools

Qualitative methods included In-Depth Interviews (IDIs), Focus Group Discussions (FGDs), and workshops with community members. Key informant interviews (KII) were also held with service providers. Development and implementation of the overall HNA was overseen by a steering committee of representatives from academia, government and the mining sector. A community champion provided local-level knowledge and support during participant recruitment and implementation. The qualitative findings for this paper are from the first two steps of the HNA framework. For the full HNA report with comprehensive methodology, refer to: http://www.wesleyresearch.org.au/wellbeing/.

Theme content for the qualitative research tools was originally developed by the qualitative research team following review of the literature and discussion with both the steering committee and local community contacts. Theme lists were developed and included perceptions of health and wellbeing at a community (IDI, FGD, KII) and service level (KII); multi-sectoral interaction and support (KII); barriers and facilitators to achieving good health (all); influences on good health (all) and perceptions of how to engage the community in health and wellbeing activities (all). FGD theme lists were further developed from preliminary findings from the quantitative survey and KIIs; for example, few survey respondents answered the open-ended questions about health and wellbeing priorities and so the FGD questions were adapted to include an emphasis on exploring the prioritisation of needs.

For the workshops, participants were asked to list and rank key health and wellbeing needs and discuss a chosen photo that represented health or wellbeing in the community. Questions were open-ended and participants were encouraged to talk about topics in their own terms. Continual reflection and debriefing occurred within the research team following each interview. Field notes assisted with reflexivity of the experiences.

### Recruitment of study participants

1. ***Stakeholder analysis and consultation with service providers and community leaders***
  A detailed mapping process identified service providers, health authorities, local governments and key community leaders in the health and community sector. Stakeholders were informed about the research project and invited to participate in a KII. Additional informants were recruited via snowball sampling and through attendance at local interagency meetings. Only the resident population was contacted for research in this study.
2. ***Community consultation with the general population***
  An expression of interest form was attached to the surveys sent out to a random sample of adults (>18 years of age) in each LGA (total = 6000) as part of the larger mixed method study. Those interested in further participation were invited to attend a FGD in their local community or an IDI over the phone. When there was clear indication of specific non-responding groups (e.g. young adults) to the survey or expression of interest, targeted stratified purposive sampling was utilised. Key informants assisted with promotion of research through email mail-outs and distribution of flyers. Middle- to older-aged community members were more likely to participate in the community FGDs and overall, females were more likely to be involved compared to males (Table 2).

**Table 2:** Qualitative primary research involved 45 key informant interviews with health and community service providers; and 11 focus group discussions and 19 individual in-depth interviews with community members across the four study sites

Region	Key informant interviews (KII) *Organisations (n)*	Focus group discussions (FGD) *Community members (n)*	Individual interviews (IDI) *Community members (n)*
Region 1	Primary care and community services (5)	Male group (3)	Male (1)
	Hospitals (4)	Female group (10)	Females (4)
	Specialised health and community services (3)	Mixed group (4; 1 male, 3 females)	
	Public health services (2)		
Regions 2&3	Specialised health and community services (11)	Male group (6)	Male (1)
	Primary care and community health services (4)	Female group (6)	Females (2)
		Mixed group (6; 1 male, 5 females)	
		Mixed group (9; 2 males, 7 females)	
Region 4	Specialised health and community services (10)	Male group (4)	Males (5)
	Primary care and community health services (3)	Female group (3)	Females (6)
	Hospitals (2)	Female group (4)	
	Government (1)	Mixed group (4; 1 male, 3 females)	
TOTAL	45	59	19

### Analysis

**Fig. 2:**
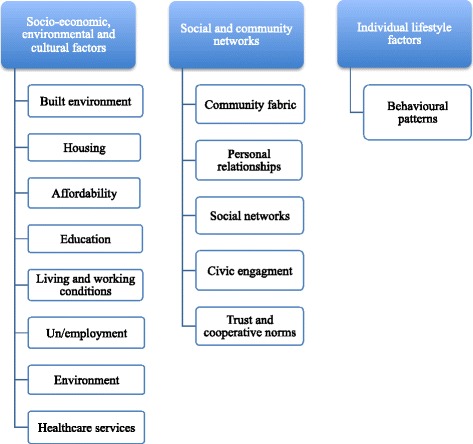
The Framework Method was developed with reference to the social determinants of health model and provided the authors with an initial structure to systematically reduce and analyse the data

Qualitative analysis was initially completed using the Framework Method to explore key themes from the FGD, IDI, KII and workshop transcriptions. The Framework Method provides an initial structure whereby the researcher can systematically reduce the data in order to analyse it. This Framework Method was guided by the social determinants of health and social capital frameworks (Fig. 2). In the first instance the first and second author analysed the data for the comprehensive HNA. The first author then coded the data again based on the emerging theme of public health and mining activity, which was then independently verified by the second author. NVivo Qualitative Software was used to analyse the data. Findings from the KIIs, IDIs and FGDs were triangulated against each other to confirm and verify findings.

### Ethical considerations

This study was granted ethics approval by the Wesley Hospital Human Research Ethics Committee, (reference number 1410). All participants were given verbal introduction to the study and provided with an information sheet to read. Participants were asked to sign a consent form if they wanted to proceed with the interviews and FGDs. Pseudonyms have been used and all other identifiable information removed for data storage and reporting.

## Results

Communities in regional Queensland faced social, economic and environmental impacts during the development phase of the CSG mine cycle. These factors were perceived to have direct and indirect effects on individual lifestyle factors such as alcohol and drug abuse, family relationships, social capital and mental health; and community-level factors including social connectedness and trust. Participants highlighted concern for sustained impacts on health and wellbeing, including how the community would cope in the ‘bust’ period; whether the community would regain its identity; how children would grow up following family-related stress during the current mining ‘boom’; and how young ex-mine employees would respond to reduced salaries outside of the mining sector.

### Socio-economic and environmental conditions

During the study period, participants in regions 1 and 4 were concerned with increasing cost of services in the community and subsequent stress and outmigration, and the perceived burgeoning division between those who benefited economically from the CSG development, and those who didn’t.

Residents in all regions commented on development of built infrastructure - most noticeably the increased availability of food outlets, liquor stores and takeaway restaurants. Availability of these options was perceived to cater for the increase in shift workers and temporary residents. Participants were concerned about the increased availability of these services in the community, and thought that young families and time-poor adults might also take advantage of convenience foods, which are often less healthy than home-cooked meals. Air travel was also an issue due to the high costs of airfares during periods when FIFO employment was at its busiest. There was concern for affordability of airfares for both leisure and to attend health and emergency medical appointments in major cities. Participants also commented on the increased number of sporting groups and clubs, but felt they were underutilised due to time constraints of shift workers. Several participants commented on the looming mining downturn and the effects this would have on demand for social and community services that had opened during the ‘boom’ to meet population growth.

Some young men with higher disposable salaries, both community members who were employed in the CSG industry and those who lived temporarily in the host communities, were often associated with antisocial conduct including alcohol-related behaviour and spending less time with family. This was pertinent in region 4. One participant was concerned for the lifestyle of some shift workers:

*“They come home, they spend an hour, have a shower and then they go to sleep because they start again the day after. And again, at the same time, link that to a low level of education and a low level of understanding, and self-awareness… drinking, constantly being with men, and having a lot of disposable income.”* Service provider, specialised community services, region 4

Participants were concerned for spill over effects in the community and what impact the short-term contracts and uncertainty in employment would have on those who had moved to the community with their families, particularly in regions 1–3. A lack of employment opportunities following the mine downturn was predicted and there was concern for the community’s ability to cope in this situation. One participant commented on the influx of people who sought work in the mining sector and remained in the community despite being unsuccessful:

*“It normally peaks, it happens in these times. We have a lot of family breakdowns. It’s normally because you know… A lot of people are saying we are an industrial city look what is happening. So we get these families who arrive thinking they will be getting these marvellous jobs on a $100,000 a year they get there with their family and realise they can’t afford the rent and there is no work for them. The family breaks down… the husband starts drinking... Drugs as well.”* Service provider, community service, region 4

CSG exploration and drilling occurred on private land and there was concern related to the disruption caused by flares, and the effects of CSG on water bores. There were issues raised relating to the environmental effects on fresh water sources in regions 2–4, which deterred participants from fishing for both recreation and consumption. CSG infrastructure also caused increased noise pollution and traffic, which affected community satisfaction with their environment and perceptions of safety.

High rental prices and poor housing availability was linked to the labour-intensive development stages and subsequent population growth, and forced many community members to move elsewhere, as summarised by this participant:

*“Community member (CM): There has been a shift in the community in the last few years around [region 1]… there’s been a lot around wellbeing and affordability too. I think there has been a lot of pressure on that just with the CSG industry in [region 1]; it’s probably put a bit of pressure on some people’s wellbeing, affordability wise… Probably not us specifically, but I have seen a lot of change around that in the community.”*

*Interviewer (I): OK and how has that impacted on people’s wellbeing would you say?*

*CM: I would say, stress.*

*I: OK. And what are people doing?*

*CM: “They are moving. They are leaving.”* Community member, region 1

Housing issues related to both the mining ‘boom and bust’ were regularly commented on across all regions. Participants commented on the surge of houses and hotels built to meet the demand during the mining boom. However, increased cost of living and housing prices strained the ability of social services to meet housing demand. Participants felt that prices had started to ‘return to normal’ leading in to the operational phase of CSG. Conversely, there was then great concern for the surplus of housing and the lack of planning by council, as described by this community member during the downturn:

*‘The councils - they’re to blame - they’re building it up all the time - how it’s going to be the greatest thing to happen to [region 3] and then they… Look at it now they’ve all left town, it was only going to be short term anyhow until they built everything and it’s all been built. There are suburbs out here with houses and houses and there’s no one in them but the fact is they’re still building them on the flood zones.’* Community member, region 3

Service providers and community members discussed the effects of living and working conditions associated with the mining sector in all regions. There was concern for the impact of shift work on families and social behaviour of mine employees in the community. Service providers commented on a marked increase in family disconnection, unbalanced lifestyles, stress and a lack of social networks for newcomers to the communities. These issues were particularly pertinent for the inner regional area 4:

*“Mental health is an increasing issue for all regional communities and I think here in particular we have problems with isolation because families move here for work and they aren’t supported; or families move here and the husbands are out, or they go out for a week or two weeks at a time, and leave what is essentially a single family, a single parent family.”* Service provider, community service, region 4

Community members and service providers commented on relationships between male mining employees and their children and felt that a lack of time spent together due to long working hours could have detrimental effects on child development. Long hours and shift work also placed pressure on mothers to carry out dual responsibilities. These issues were traded off against higher wages afforded to shift workers and the benefits of having financial security.

*“Shift work. I think that there is some comorbidities that develop amongst the communities that is very much related to long hours, separation from family, unnatural working hours… Even though people who do continual shift work begin to see that as normal, in actual fact it deters that negative impact on us as you know, people. I think you see stress, depression, obesity, diabetes, and dysfunctional relationships.”* Service provider, tertiary services, region 4

### Individual lifestyle factors

Linked to an increase in a male-dominated environment and higher disposable incomes, participants perceived an excessive use of illicit drugs and alcohol in the community. References to drugs and alcohol were particularly salient in region 4, with concerns for the increased availability of drugs in regional communities:

*“I: What would be the most pressing health need for the community or for people in your area?”*

*CM: Probably the drinking would be a big thing.*

*I: The drinking - ok - and any other things?*

*CM: Ah in some particular mining camps the drugs are getting in there now.*

*I: And is that having much sort of spill out into the community?*

*CM: “From time to time there is and there has been an increase in the drug raids happening in and around town due to mining people getting hold, of bringing in drugs and then selling them.”* Community member, region 4

All communities were concerned for the effects of excessive drug and alcohol consumption among young mine employees. Service providers in region 4 linked the sudden demand for domestic violence support services to the behaviours of partners’ who worked in the mine industry. Participants in region 4 mentioned anti-social behaviour in the town centre and insecurity felt by female residents alone in the town at night. It was perceived that these behaviours in the community were unwelcoming to other newcomers outside of the mine workforce.

*“The other thing some of the local ones, I won’t say all of them because I know they all don’t do it but some of the local ones who have scored jobs in the industry have been on outrageous wages and what are they doing with those wages, I only have to go I won’t tell you where I have to go to buy cocaine and methamphetamine and whatever, but it is so easy to get and these people have a disposable income and they’re young they’ve got no common sense that they’re not old enough to have that yet.”* Community member, region 2

Residents in region 4 cited the influx of the mine workforce in pubs and high purchasing rates of alcohol in the community, particularly during poor weather when employees were unable to work. It was felt that workers had little else to do in the community. Participants in regions 2 and 4 felt that the traditional, family-oriented pub culture of the community had dissipated because of expensive prices and over-crowding by the mine workforce. In region 4, participants commented on the cultural changes and the lack of social nightlife in the main town:

*“I think probably there are just a number of groups and they interact at different times out of need. I think that springs back to the basic social lifestyle, which is around shift work. Like you know, this town it’s really busy, you can go in at 9’o clock on a Friday and everyone is just about disappeared apart from the nightclubs. You know, it’s just an unspoken rule because people are up travelling at about 3.30 am/ 4am. So, because of that, it doesn’t evoke community as much. People aren’t sitting around until late at night, just enjoying themselves down town because people have gone.”* Service provider, tertiary services, region 4

### Social capital and community networks

Communities in each region experienced rapid population growth during the study period. Several references were made to the transient nature of the population –interstate, multi-state and overseas migration led to an impact on community culture, particularly because of the impermanent nature of newcomers and contrast with more traditional and regional community values. One participant recalls the traditional clothing often worn by country Australians, which was associated with farming and agricultural lifestyles, and how this is less prevalent in the community now.

*“There is a change in the values. And there is a lower density of Akubra hats and moleskin [trousers] as you go down the street; it’s more reflective gear and every second vehicle has a flag on it. And that’s a whole different culture to what was here.”* Community member, region 1

Participants discussed the impact of population growth and CSG related infrastructure on social isolation in regions 1–3. Residents withdrew from services in town because of the changing nature, and this was a particular concern given that many residents lived on rural farms with few socialising opportunities outside of their visits to town.

*“I think too, with the influx of the gas, you know we call them ‘glow worms’ - with the big bright shirts, they are everywhere you go in the coffee shops, in the restaurants, everywhere you go. You walk down the street and the vehicles with their little flags. You think ‘ohhh gosh’, just from a visual point of view, that just impacts. And the traffic got a lot more. I mean there are positive and there are negatives, but from a community point of view the ones that have been here longer term have probably withdrawn from the services, they don’t feel so connected. Like people can say oh it’s not the same place I moved to.”* Service provider, specialised services, region 1

Long-term residents felt that newcomers did not want to contribute to the community. Conversely, newcomers felt isolated and some felt they weren’t welcomed in the ‘cliquey’ town. One participant commented on feeling unwelcome in the community due to temporary residence on a street with other FIFO employees.

*“Being isolated as a worker like I’m a – they call workers like myself a townie. A townie is somebody who works in town, they’re here for 3-5 years usually or shorter and I’m actually not part of the community, so some community events some church events they don’t always make workers like myself feel particularly welcome because they know they’re only here for a short period of time. So that’s difficult - a bit of a cliquey town. There’s lots of wealthy land owners as well as workers in town… that probably comes back to isolation and not having sort of a connection to this community because they don’t have family.”* Community member, region 1

Newcomers were often described as transient people who were ‘coming for the economy with no intention to stay’. Community members in region 4 mentioned the under-utilised cemetery as an example of the few people who stayed permanently to retire and live the rest of their life in the region.

Participants in region 1 linked poor community wellbeing to inadequate engagement and communication with the mining sector; Participants were also concerned for the level of community reliance on the mining sector. They felt that the relationship between community members and the sector could be improved, as described by these participants:

*“CM 1: You know so we’re a corporate town, we’ve been ‘corporatised’ and now I think people are getting it in their head that they’re de-culturising and that if the town wants something, well the resource company will fork out the money and we’ll just leave it up to them and I think a lot of the young people are seeing that. They’re seeing that the school - you don’t have to work for it. Yeah that’s right the money will just come from them. You’re not seeing, like I was very offended to see those signs on the school on every side of the school there’s a [mining company] sign and I was thinking now hang on when the brothel comes to town, are they going to be allowed to sponsor the school and put their signs up and what about the hundreds and hundreds of parents over the years that have contributed to that school so where’s their name around the oval.”*

*I: I’m just trying to make sure we add it to this, what’s the wellbeing need there?*

*CM 1: “To keep more community engagement.”* Community members, region 2

## Discussion

The findings in this study support anecdotal evidence of broader health concerns arising from nearby CSG development beyond direct physical health impacts. Communities in this study perceived there to be both direct and indirect impacts of CSG development at both an individual and community level. Outer regional communities (regions 1–3) discussed the effects of mining activity on the fabric of their town and community, whereas the inner regional community (region 4) that had a longer history of industrial activity discussed the impacts on families and individual health and wellbeing. Region 1 is much larger than regions 2–4 but with a much smaller population, which could explain the prominence of community-level health and wellbeing impacts of mining. Region 4 had a greater transient and a younger population, which could explain the focus on individual-level health wellbeing needs [19]. Regions 1–3 were predominantly agricultural regions, which could explain why community members were concerned for the stages following construction, when the population would decrease as quickly as it increased, along with employment opportunities and demand for services. Region 1–3 may be more sensitive to the impacts of CSG development because they are smaller and less developed than region 4. The density and geographical size of the community and its previous experience with mining or other industries is predicted to influence the magnitude of impacts felt [20].

### Socio-economic and environmental conditions

The Queensland Government described CSG and LNG development as a ‘once in a generation opportunity providing jobs, energy security and prosperity for citizens’ [21]. This study demonstrated how the stage of mining activity and subsequent local economic fluctuations affected the social and environmental fabric, which in turn had consequences for health and wellbeing needs at individual and community levels. The ‘rapid’ nature of CSG development is perhaps a reflection of the labour-intensive development stages, and the short-term impacts this had on the community. It would be valuable to study the effects on health and wellbeing during the consequent stages of mining, to ascertain if the results from this study are unique to the development/pre-construction phase.

A key source of economy for regional Queensland is farming and agriculture. CSG activity commonly occurs on active farms and grazing properties which provides increased opportunity for human interaction and conflict [1]. In the study sites and in the wider literature, CSG development was perceived to disproportionately affect farmers [22]. CSG development involves large water supply usage, environmental disruption and overlap with existing farmland. These conditions could contribute to financial and environmental concerns and influence stress and anxiety levels in an already vulnerable population [23]. Apprehension related to the increased cost of living and uncertainty was thought to force residents out of the communities in search for more affordable living. Economic insecurity can negatively affect mental health [24]. Local mental health services in mining-affected communities need to be aware of the potential triggers during ‘boom’ periods in order to effectively target services, and monitor and respond to needs.

During the study period, mining communities in regional Queensland experienced significant changes to the built environment and natural landscape, including rapid growth of takeaway and fast food outlets to meet population demands. The public health implications relating to the marked increase in these services is a concern, considering the higher rates of overweight and obesity in rural areas compared to major cities [19]. Increased availability of fast food and takeaway outlets is linked to increased prevalence of obesity in young Australian adults [25]. CSG development utilises a large amount of land due to the multitude of dispersed gas wells, difficult road access, pipelines, and processing plants and dams [1]. In concentrated community centres, this has led to concerns around traffic, volume of activity and destruction to the natural environment, impacting on community wellbeing. Local government is responsible for planning and managing such changes, but unforeseen impacts of the fast-growing and new industry may have contributed to negative community perceptions. Ex-gas mining communities in the US have been branded as ‘ghost towns’ and ‘contaminated communities’, reflecting the exodus of people following the mine downturn and little incentive to stay in such altered environments [26]. Evidence-informed planning and communication between local government, mining companies and the public is integral to ensure that long-term effects in the community are mitigated.

### Living and working conditions

Social conflict, substance abuse and domestic violence has been linked to the social ‘costs’ of CSG development and are often considered tertiary socio-economic impacts [12]. These issues were key concerns in the study regions, particularly the social impacts of shift work on families and partners. A lack of understanding of the duration of mine activity led to some tension between longer-term community members and the temporary residents who arrived to work for the mining companies. Some temporary residents felt isolated and unwelcome, feelings of which can lead to poor health and wellbeing and a lack of community cohesion [23].

### Individual lifestyle factors

The communities were concerned that working conditions, particularly for young males, led to anti-social behaviour in the community and excessive drug and alcohol abuse. These risky lifestyle behaviours can have significant impacts on mental health and long-term chronic diseases like lung cancer and liver disease. The working conditions of mine employees and potential for risky lifestyle beahviours is often referred to as a socio-economic product of the ‘boom town effect’ [12, 27]. There has been little research on the implications of CSG development on women but communities in this study were concerned for the impact of working conditions on families and the effects of social isolation on women. There was an identified need for improved social services to support women in these situations.

### Social capital and community networks

Social capital represents social connections and the benefits they generate. Social capital can be sourced at an individual (e.g. family support) or wider collective level (e.g. volunteering) [28]. The framework used in the analysis demonstrated the link between CSG development and community fabric, neighbourhood interactions, community satisfaction, trust and cooperative norms.

It was important to community members to understand what was happening in their communities. As CSG is a relatively new industry there was significant uncertainty and anxiety around the unknown effects. Brashier [2011] stated that community reaction to mining development spans four stages: enthusiasm in the initial stages; followed by uncertainty; then panic and finally, adaptation [29]. The term ‘solastalgia’ has been coined to describe the melancholy felt following the unwelcome change in one’s community and is often used in the CSG development context [30]. At a community level, there is a responsibility of local government to provide evidence, transparency and awareness around the CSG mining process to mitigate negative reactions. It is possible that perceived impacts of CSG development on health and wellbeing may reflect an unavailability of reliable sources, inadequate community consultation and a possible reliance on media for information.

‘Community resilience’ is a term often used in the context of mining and regional communities, and it is even quoted as a local government objective [31]. Resilience can be defined as responding to changes in one’s community with a view to reinstate, maintain and enhance community wellbeing [31]. A key focus of research in this context is being able to provide evidence that supports communities in preparing for the local effects of CSG development rather that experiencing uncertainty and disruption.

### Public health and policy implications

Extraction of CSG can occur alongside communities for over a decade. There is obvious concern that a lack of assessment of ongoing and cumulative health impacts leads to mining projects being carried out without a thorough understanding of the consequences for host communities [8]. In Australia, this is evidenced by submissions of concern by leading public health organisations to the NSW independent enquiry and commentaries from prominent academics in the field on NSW CSG development in 2013 [7, 8, 32, 33]. There is currently a lack of cohesion in identifying what health and wellbeing outcomes should be considered when examining population-level impacts of mining, and which stakeholders should be held accountable. Research demonstrates that impacts of CSG development stages relate to social, economic and environmental factors that can affect an individual beyond the state of physical health [12, 34]. Furthermore, evidence points to community—level health and wellbeing impacts that, although harder to measure due to the myriad of possible causes, merit attention (Table 3).

**Table 3:** Summary of key findings and recommendations

Key Findings	Context	Recommendation
CSG mining during development stage has implications for the social determinants of health (SDoH) and health and wellbeing outcomes	Direct and indirect impacts both at individual and community level	Potential impacts of CSG mining could incorporate standardised assessment of SDoH at individual and community level, with acknowledgment that setting (e.g. level of remoteness can affect magnitude of outcomes; avoid ‘one size fits all’ approach
Density and remoteness affects magnitude and type of impacts felt	Inner regional experienced more individual level impacts vs outer regional which experienced more community level impacts	
Effects on health and wellbeing may vary with the stages of CSG mining	Lack of assessment of ongoing and cumulative health impacts through the stages	Monitor health and wellbeing over time to enable evidence-informed planning and response to fluctuating demands
Lack of community understanding of CSG timeline and local impacts	Insecurity, lack of trust and concern for the future following completion of CSG mining could exacerbate negative perceptions	Communication of short and long term impacts is imperative alongside effective mitigation and planning
Population level studies are effective to highlight opportunities for targeted research	Groups that might be disproportionately affected by CSG included farmers, young families and women	Targeted research to determine what services are in place or required to meet temporary or longer term needs
Measuring and responding to the impacts of a mining project is not the responsibility of the mining company alone	Assessments should focus on the population, not the project, in order to uncover health and wellbeing outcomes that may not have otherwise been captured	A partnership approach involving local government, communities, research institutes, mining companies and social and health organisations is imperative

There is still much debate and uncertainty around the best tools to measure health and wellbeing impacts in CSG development regions. In the health sector, proxies to determine health impacts include assessing hospitalisation rates and access to health services [6]. Historically, the potential impacts of mining was assessed through health, environmental and/or social impact assessments. According to the International Finance Corporation guidelines, a Health Impact Assessment (HIA) involves the collection and evaluation of baseline data and subsequent risk assessment, and the outcome should include an action plan that addresses the risks previously identified and a monitoring and evaluation strategy [35]. HIAs should incorporate tools to capture broader health and wellbeing outcomes under the social determinants of health framework, and outcomes should be monitored at several points throughout the mining lifecycle.

This study highlights the importance of gaining community views to understand broader health implications of CSG development – the study revealed interesting associations between mining activity and both individual and community level wellbeing. The findings demonstrate the importance of engaging with communities to identify issues throughout the mine cycle, and to use primary qualitative research to gain a deeper understanding of some of the drivers of poor health and wellbeing.

The prevalence of sex work was not mentioned by participants in any of the regions. Sex work in mine settings is a relatively well known occurrence and there are legalised brothels in Queensland, potentially making it a ‘non-issue’ in these communities [36]. It is also possible that with the discretion around sex work, its occurrence may not have been obvious to participants, or they simply did not consider it as a health or wellbeing need in this region.

This exploratory study highlighted potentially vulnerable groups that may be affected differentially by CSG development, including women and farmers. It is also important to consider whether effects on health and wellbeing differ between migrant populations and permanent residents. Further research could involve assessing health and wellbeing needs of specific groups using the HNA approach. What used to be an ‘iron triangle’ of government, industry and science needs to incorporate civil society, media and broader stakeholders to enable monitoring, prediction and management of cumulative impacts at a local community-level, and at all stages of mine activity [10].

CSG mining is often referred to as ‘rapid’ due to the growth of the industry over a short time, labour intensive yet relatively short development phase, and lack of understanding of the possible implications in the local community. It is imperative to understand the context within which CSG mining occurs to predict and control health and wellbeing impacts. More populated communities with existing mining and industry may be less likely to ‘feel’ impacts of development stages. As our understanding improves of the implications of CSG mining and communities are better prepared for the development stage, effects may become less damaging.

Individually regulating impacts on health and wellbeing is virtually impossible because multiple companies often work in one region and impacts cannot be solely attributed to a particular mining activity [1]. There is also variation in the institutional frameworks that define what health and social assessments must be conducted as part of a mining company’s corporate social responsibility, and the findings are often not available in the public domain. HNAs focus on the population rather than a project, and therefore encompass broader health and wellbeing needs and, because of this, HNAs take the responsibility of implementation away from being solely that of the mining sector towards a joint obligation with communities, local government, research institutes and social and health organisations. HNAs are implemented with a partnership approach and significant community involvement, and the outcomes are useful for policy to inform regional and local strategic planning.

## Limitations

It is important to note that the HNA process would not have enabled the possible *positive* health implications of mining activity to be revealed, because the aim of the assessment was to determine *needs*. Older women were more likely to take part in the qualitative research and there is a risk of bias and misrepresentation in perceptions because of this. Furthermore, it was known to the research team that the study sites had experienced significant consultation fatigue due to other social, economic and health-related research in the area, which may have contributed to the small population sampled. This corroborates the need for a unified approach to measure, manage and respond to health and wellbeing impacts of CSG development. It is also preferable to gain perceptions from a heterogeneous cross-section of the population with a broader age range than that of this study.

## Conclusion

There is evidence of indirect and long-term health and wellbeing implications of living in proximity to CSG development. How communities respond to the boom, post-boom transition and ‘bust’ of CSG development is important for government, the mining sector and the scientific community. The findings from this study may inform health service planning in regions affected by CSG development and provide the mining sector in regional Queensland with evidence from which to develop social responsibility programs that encompass health, social, economic and environmental assessments that more accurately reflect the needs of the community.

HNAs are a valuable tool for determining cumulative outcomes and needs and operate at population-level rather than project-level. Measuring wellbeing in addition to health provides a more realistic profile of the community. It is recommended that further research is conducted at all stages of the CSG mine cycle to determine trends in health and wellbeing and appropriate responses.

## Abbreviations

ARIA: Accessibility/ Remoteness Index of Australia
CM: Community member
CSG: Coal seam gas
DIDO: Drive-in-drive-out
FGD: Focus group discussion
FIFO: Fly-in-fly-out
HIA: Health impact assessment
HNA: Health needs assessment
I: Interviewer
IDI: In-depth interview
KII: Key informant interviews
LGA: Local government area
LNG: Liquefied natural gas
SDoH: Social determinants of health
